# Fractionated Therapy of HER2-Expressing Breast and Ovarian Cancer Xenografts in Mice with Targeted Alpha Emitting ^227^Th-DOTA-p-benzyl-trastuzumab

**DOI:** 10.1371/journal.pone.0042345

**Published:** 2012-08-03

**Authors:** Helen Heyerdahl, Nasir Abbas, Ellen Mengshoel Brevik, Camilla Mollatt, Jostein Dahle

**Affiliations:** 1 Department of Radiation Biology, Institute for Cancer Research, Oslo University, Hospital - The Norwegian Radium Hospital, Oslo, Norway; 2 Department of Research and Development, Algeta ASA, Oslo, Norway; 3 Department of Research and Development, Nordic Nanovector AS, Oslo, Norway; National Institute of Health, United States of America

## Abstract

**Background:**

The aim of this study was to investigate therapeutic efficacy and normal tissue toxicity of single dosage and fractionated targeted alpha therapy (TAT) in mice with HER2-expressing breast and ovarian cancer xenografts using the low dose rate radioimmunoconjugate ^227^Th-DOTA-*p*-benzyl-trastuzumab.

**Methodology/Principal Findings:**

Nude mice carrying HER2-overexpressing subcutaneous SKOV-3 or SKBR-3 xenografts were treated with 1000 kBq/kg ^227^Th-trastuzumab as single injection or four injections of 250 kBq/kg with intervals of 4–5 days, 2 weeks, or 4 weeks. Control animals were treated with normal saline or unlabeled trastuzumab. In SKOV-3 xenografts tumor growth to 10-fold size was delayed (p<0.01) and survival with tumor diameter less than 16 mm was prolonged (p<0.05) in all TAT groups compared to the control groups. No statistically significant differences were seen among the treated groups. In SKBR-3 xenografts tumor growth to 10-fold size was delayed in the single injection and 4–5 days interval groups (p<0.001) and all except the 4 weeks interval TAT group showed improved survival to the control groups (p<0.05). Toxicity was assessed by blood cell counts, clinical chemistry measurements and body weight. Transient reduction in white blood cells was seen for the single injection and 4–5 days interval groups (p<0.05). No significant changes were seen in red blood cells, platelets or clinical chemistry parameters. Survival without life threatening loss of body weight was significantly prolonged in 4 weeks interval group compared to single injection group (p<0.05) for SKOV-3 animals and in 2 weeks interval group compared with the 4–5 days interval groups (p<0.05) for SKBR-3 animals.

**Conclusions/Significance:**

The same concentration of radioactivity split into several fractions may improve toxicity of ^227^Th-radioimmunotherapy while the therapeutic effect is maintained. Thus, it might be possible to increase the cumulative absorbed radiation dose to tumor with acceptable toxicity by fractionation of the dosage.

## Introduction

Breast and ovarian cancer caused an estimated 590 000 cancer deaths worldwide in 2008 [1]. Overall survival rates for patients with disseminated cancers are poor despite improvements in therapy methods [2], [3]. The human epidermal growth factor receptor 2 (HER2, also known as ErbB2/c-erbB2/HER2-neu), a transmembrane receptor tyrosine kinase, is overexpressed in 25–30% of breast cancers [4] and 6–50% of epithelial ovarian cancers [5]. The humanized monoclonal antibody (mAb) trastuzumab (Herceptin®, Roche) targets HER2 and gained FDA approval in 2008 for treatment of HER2-overexpressing metastatic breast cancer. However, the efficacy of HER2 therapy alone is limited in advanced cancers and resistance is developed in most patients within a year after start of therapy without significantly downregulating the numbers of HER2 antigens [6]. Resistance often develops despite sustained HER2 expression [7]. The persistent exposure of HER2 on the tumor cells may be utilized for targeting of a pay-load, including cytotoxic agents and radionuclides.

One advantage of utilizing a radionuclide compared to a cytotoxic agent for tumor targeting is that fewer antigens per cell need to be targeted to obtain cytotoxicity, meaning that patients with lower antigen expression can be treated. In addition, resistance is not likely to occur [8].

**Figure 1:**
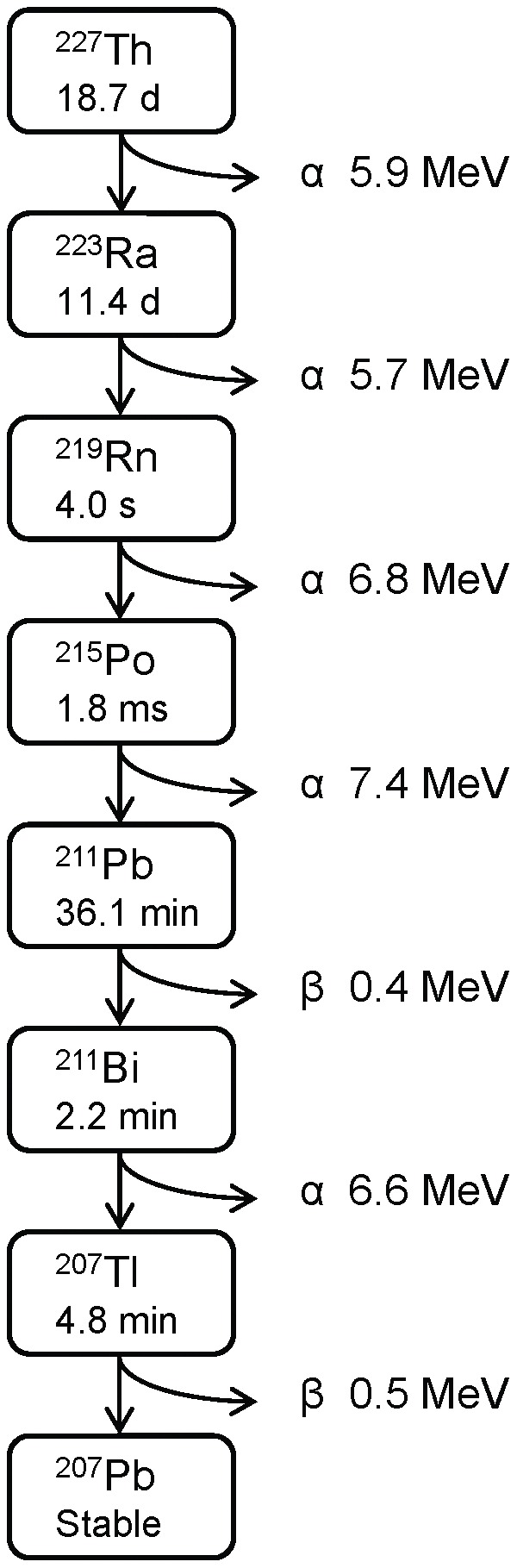
Th-227 decay scheme.

Radioimmunotherapy with alpha-particle emitting radionuclides (targeted alpha therapy, TAT) was first reported in clinical trials in 1999 [9]. TAT utilizes strongly cytotoxic high linear-energy-transfer (LET) alpha-particles to kill cancer cells. The damage on normal tissues is minimal, due to the short range in tissue (less than100 µm). Thorium-227 (^227^Th) is an interesting candidate for TAT of disseminated cancer due to its long half life and its favorable chemical properties allowing ^227^Th to be stably chelated and conjugated to monoclonal antibodies (mAbs) [10]. Thorium-227 decays with a half-life of 18.7 days, emitting an alpha-particle of 5.9 MeV. A cascade of decays follows, resulting in emission of another four alpha-particles with mean energy of 6.6 MeV before ending with the stable nuclide ^207^Pb (Figure 1).

TAT of hematological tumors has been more successful than TAT of solid tumors. Solid tumors are generally less sensitive to radiation and are more difficult to target due to macromolecule transport barriers. However, it has been shown in previous studies that ^227^Th conjugated to trastuzumab could specifically kill HER2-overexpressing cancer cells *in vitro* and had a therapeutic effect on HER2-overexpressing subcutaneous tumor xenografts [11], [12], [13]. Although an anti-tumor effect was seen, the maximum tolerated dosage was not reached in these studies. However, in a previous study with ^227^Th-rituximab we showed that the maximum tolerated dosage for Balb/c mice was between 600 and 1000 kBq/kg [14]. The highest dosage resulted in myelotoxicity and weight loss.

Giving more than one dose is expected to result in more homogeneous distribution of the mAb in the tumor compared to giving a single administration [15], [16]. From previous in vitro studies we have seen that the HER2 antigen expression was not evenly distributed among the cells [11]. The likelihood of targeting and hence killing high-expressing cells might be larger than targeting neighboring cells, or at least the relative uptake is larger. The distribution of a second dose may be improved due to increased accessibility and permeability resulting from the first dose of RIT, e.g. through increased leakiness of the vessels [17]. Most importantly, fractionation of the dose will prolong the treatment period and enable giving a larger accumulated dose of radioactivity, improving the therapeutic effect. For example, the severity of white blood cell (WBC) deficiency may be reduced by allowing a recovery period sufficient for production of new WBCs following each dosage. Therefore, the optimal time period between administrations depends on both therapeutic and toxic effects.

The objective of the present study was to compare the toxicity and therapeutic efficacy of ^227^Th-trastuzumab when giving four injections separated by different time periods, compared to one single injection known to be efficacious and close to maximum tolerated dose (MTD).

## Materials and Methods

### Preparation of ^227^Th-DOTA-p-benzyl-trastuzumab

Thorium-227 and ^227^Th-DOTA-*p*-benzyl-trastuzumab (^227^Th-trastuzumab) were prepared by Algeta ASA, Oslo, Norway [18]. Trastuzumab was conjugated with *p*-SCN-Bn-DOTA in sodium borate buffer (pH 9) at 37°C over night. The DOTA-Bn-trastuzumab conjugate was purified on an Amicon spin filter unit (Millipore, Cork, Ireland), diluted with 0.9% NaCl and freeze-dried before storage at −18°C. Radiolabeling with ^227^Th was performed with 1 mg DOTA-Bn-trastuzumab conjugate dissolved in sodium acetate buffer (pH 5.5), which was added about 4 MBq of newly purified ^227^Th in 0.01 M HCl. The labeling reaction was conducted at room temperature/2 days or at 60°C/30 min in a thermomixer (Eppendorf AG, Hamburg, Germany). The reaction mixture was added 10 µl saturated DTPA, and the radioimmunoconjugate was purified twice on a NAP5 column (GE Healthcare Ltd, Little Chalfont Buckinghamshire, UK) using PBS as running buffer. The radiolabelling efficiency and purity of ^227^Th-trastuzumab were determined using a GEM15 HPGe-detector (Ortec, Oak Ridge, Tennessee, USA), typically yielding 70–85% of ^227^Th-trastuzumab in >99% radiochemical purity (^227^Th vs ^223^Ra).

### Specific Activity and Immunoreactive Fraction (IRF)

The IRF was determined as previously described [13]. IRFs were in the range 39 to 83%; batches of radioimmunoconjugates with IRF below 46% were only used at two occasions in the fractionated SKBR group with interval 4–5 days. The single-point IRF method used under-estimates the IRF compared to more elaborate methods like the one published by Lindmo et al. [19]. Specific activities were in the range 800–3000 Bq/µg.

### Ethics Information

All procedures and experiments involving animals in this study were performed in accordance with the European Convention for the Protection of Vertebrate Animals used for Experimental and other Scientific Purposes. The protocol was approved by the Norwegian Animal Research Authority (Permit ID: 1547).

### Animals and Xenografts

Institutionally bred, 8–19 weeks old female Balb/c nu/nu (NCR) mice with body weights in the range 18–31 g at the start of the experiment were used. The animals were maintained under pathogen-free conditions, and food and water were supplied ad libitum. Mice were anesthetized with s.c. injection of 100 µl Zoletil mix (tiletamine/zolaxepam from Virbac S.A., Carros, France added xylazin and butorphanol from Intervet International B.V., Boxmeer, Holland) diluted 1∶5 in sterile water prior to bilateral subcutaneous implantation of 2 mm^3^ pieces of tumor tissue on the rear flanks. Mice were ear-tagged and followed individually throughout the study.

Tumor xenografts originated from human HER2-overexpressing breast cancer (SKBR-3) and ovarian cancer (SKOV-3) cells from the American Type Culture Collection (ATCC, Manassas, VA, USA). In this study only xenografts having undergone more than 10 passages in carrier mice were used.

### Therapy Groups

Two to four weeks after implantation mice with tumor diameters in the range 3.5–8.5 mm were randomly assigned to therapy groups. Only mice with growing tumors were included in the study. Control mice were given either 4 injections of 5 µg unlabeled trastuzumab in 100 µl PBS or 100 µl 0.9% NaCl in the tail vein. In the radioimmunotherapy groups, mice were given 1000 kBq/kg ^227^Th-trastuzumab as a single injection or 4 injections of 250 kBq/kg ^227^Th-trastuzumab.

In total, 145 tumors in 107 mice were included in the study, 8–12 animals in each therapy group. In addition 10 control mice without tumor were given a single injection of 0.9% NaCl. Animals with open lesions on tumors occurring during the course of the study were excluded from analysis.

### Tumor Growth Delay and Body Weight Measurements

Tumor size and animal weight were measured three times a week for up to three weeks after last injection; thereafter twice weekly unless more frequent inspection needed. Ellipsoid shapes were assumed and tumor volumes were calculated as: V = (a^2^·b)/2 where a and b represent shortest and longest perpendicular tumor diameters, respectively. Mice were euthanized by cervical dislocation if largest tumor diameter approached 20 mm, loss of body weight was considered life threatening (in this study defined as a decrease by more than 15% from maximum tumor free body weight), or animals otherwise showed symptoms of disease and discomfort.

Therapeutic efficacy was assessed by delay in tumor growth. Delay was assessed by different methods. Growth curves were made based on average normalized tumor volumes within each therapy group. Normalization of tumor volumes was done by assigning volumes at day 0 to the average tumor volume at day 0, within the experiment and across therapy groups. The individual tumor volumes at termination were kept unchanged in the mean volume calculations until the last animal in the therapy group was sacrificed. Time to tenfold increase from initial tumor volumes at day 0, and time to largest tumor diameters reached 16 mm were also assessed by survival analysis.

### Blood Sampling for Evaluation of Toxicity

Prior to start of therapy and thereafter every 3 weeks for up to 18 weeks, 100 µl blood was collected from vena saphena lateralis in 0.5 ml EDTA coated tubes (BD Microtainer K2E tubes, Becton, Dickinson and Company, Franklin Lakes, NJ, USA). Blood samples were analyzed using an automated hematology analyzer (Scil Vet abc animal blood counter, Horiba ABX, Montpellier, France) and white blood cells, red blood cells, and platelets were monitored. At time of animal termination blood for clinical chemistry analysis was collected by heart puncture during sevoflurane anesthesia (Sevoflurane; Abbott, Abbott Park, IL, USA). Approximately 0.4 ml blood was collected in 0.4 ml lithium heparin coated tubes (BD Microtainer LH tubes, Becton, Dickinson and Company, Franklin Lakes, NJ, USA). Clinical chemistry analyses were performed using Reflovet Plus analyzer and Reflotron reagent strips (Roche Diagnostics GmbH, Mannheim, Germany). Blood concentrations of urea and the liver enzymes aspartate aminotransferase (AST), alanine aminotransferase (ALT), and total alkaline phosphatase (ALP) were measured.

### Dosimetry Calculations

Theoretical dose rates to tumor, femur and blood from the different fractionation schemes were calculated based on single injection biodistribution data from the two tumor models previously published using 400 kBq/kg body weight [12], [13]. Injected activity was normalized to 250 and 1000 kBq/kg body weight. The following formula was used, assuming uniform distribution of radionuclides in the tissues:


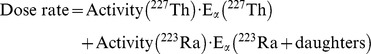


where E_α_(^227^Th) = 5.9 MeV and E_α_(^223^Ra + daughters) = 26.4 MeV [20]. The absorbed dose to organ was calculated by integrating the area under the dose rate curve from injection to time point in question. When calculating dose from fractionated injections, dose rate curves for each new injection added were assumed to equal the single injection dose rate curve.

### Statistical Analysis

Time to 10-doubling of tumor size, time to largest tumor size reached 16 mm and time to 15% loss of tumor free body weight among the different treatment groups were compared by Mantel-Cox Log Rank test using SPSS version 16.0 (SPSS Inc. Chicago, IL, USA). Kaplan-Meyer survival plots were made based on tumor size and weight loss using SigmaPlot version 11.0 (Systat Software Inc, San Jose, CA, USA). Blood cell counts and clinical chemistry parameters were compared by one-way ANOVA on Ranks and Dunn’s and Holm-Sidak Pairwise Multiple Comparison tests in SigmaPlot.

## Results

### Tumor Growth Delay

**Figure 2:**
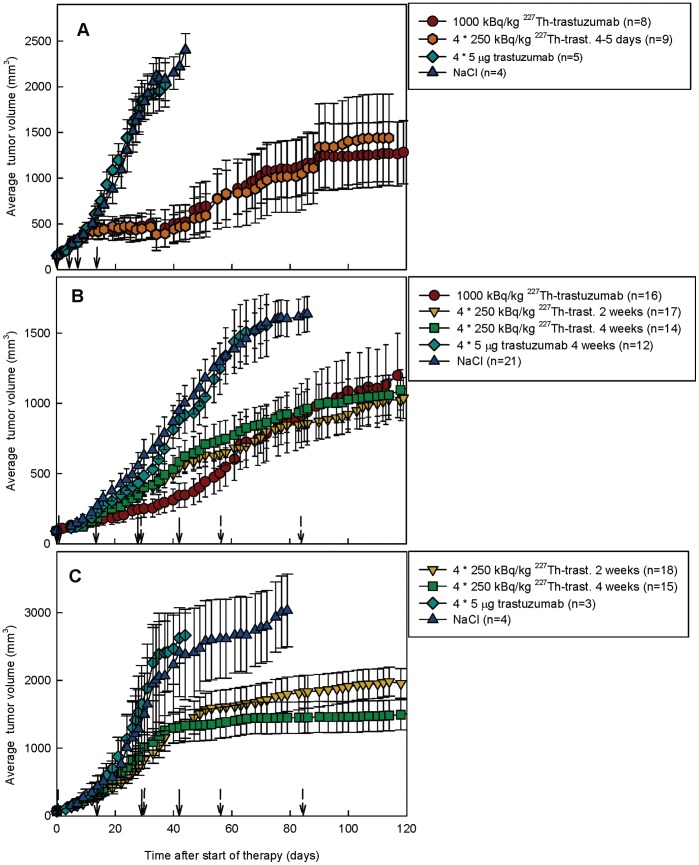
Normalized average tumor volumes as function of time. Averages of normalized tumor volumes for pilot experiment in SKBR-3 tumor xenografts (A) and further experiments in SKOV-3 (B) and SKBR-3 (C) tumor-carrying mice as function of days after start of therapy. Normalization was done by assigning all individual volumes at day 0 to the average tumor volume within each experiment. Tumor volumes at day of termination for each mouse were kept in the analysis until all mice in the therapy group were killed. Error bars are standard error.

**Figure 3:**
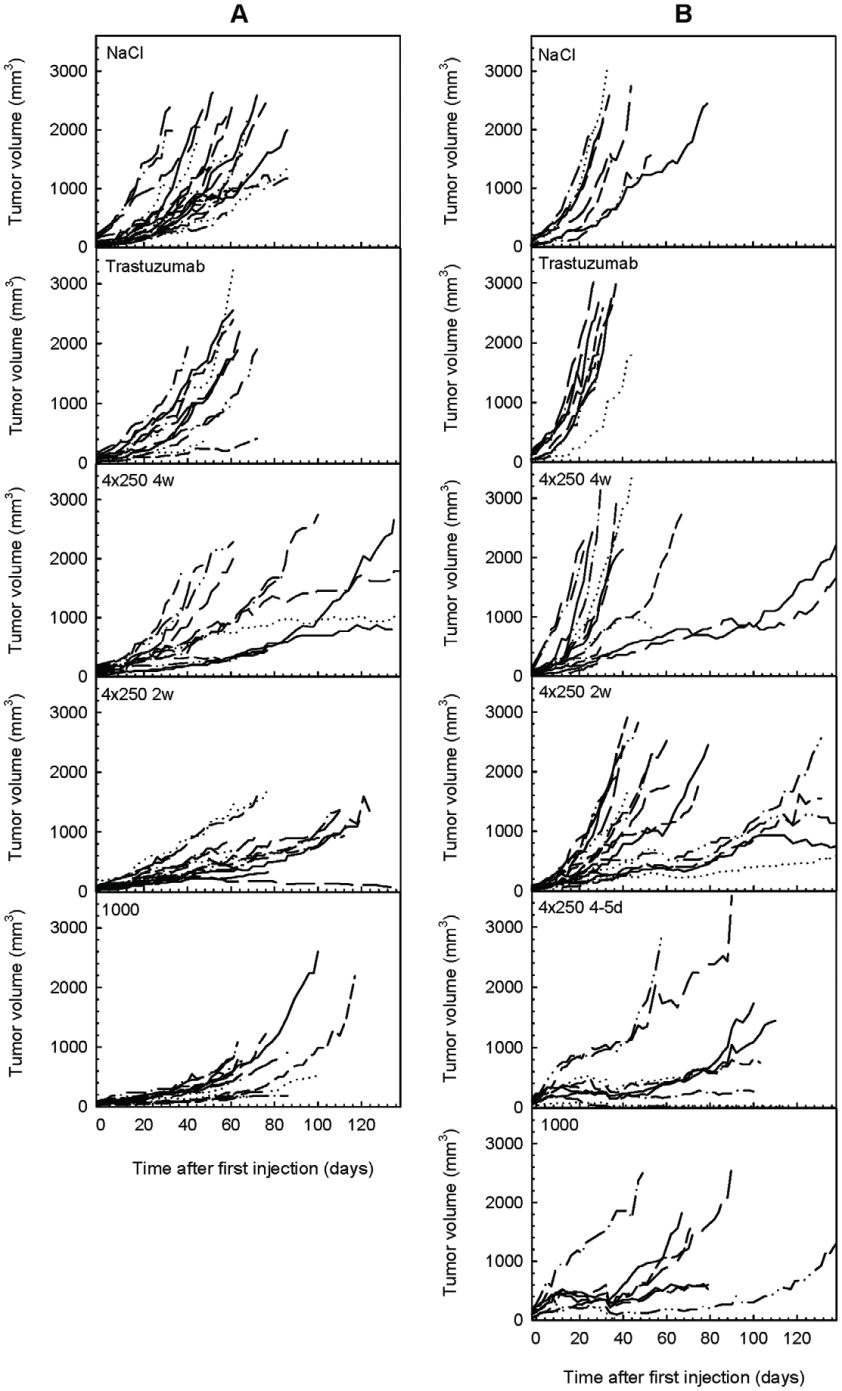
Individual tumor volumes as function of time. Individual raw data measurements of tumor volumes for SKOV-3 (A) and SKBR-3 (B) xenografts in mice. Panel labels: Groups labeled 1000 and 4×250 are given ^227^Th-trastuzumab at dosages of 1000 kBq/kg as single injection or 4 · 250 kBq/kg at intervals marked (d  =  days or w  =  weeks).

Response to radioimmunotherapy was assessed by comparing tumor growth in xenografted mice after treatment with ^227^Th-trastuzumab with growth after treatment with saline or unlabeled trastuzumab. A pilot study was done in mice xenografted with SKBR-3 tumor by administration of 1000 kBq/kg ^227^Th-trastuzumab as single injection or as 4 injections of 250 kBq/kg ^227^Th-trastuzumab separated by 4–5 days (Figure 2A). Based on results using these dosages a larger study was initiated, introducing the more slowly growing tumor line SKOV-3 and longer therapy intervals of 2 and 4 weeks. Normalized average tumor volumes and individual tumor raw data as function of days after onset of therapy are shown in Figures 2 and 3, whereas time to 10-fold increases of tumor volumes and growth delay is given in Table 1.

**Table 1:** Therapeutic effect of single injection and multiple injections of ^227^Th-trastuzumab.

Tumor volume		SKOV-3			SKBR-3		
Treatment group	Therapy interval	n*	Time†	Growth delay‡	n*	Time†	Growth delay‡
1000 kBq/kg ^227^Th-trastuzumab	–	9 (16)	72±3§,∥	27±5	2 (8)	116±14§,∥	90±14
4×250 kBq/kg ^227^Th-trastuzumab	4–5 days	–	–	–	4 (9)	98±7§,∥	73±7
4×250 kBq/kg ^227^Th-trastuzumab	2 weeks	10 (17)	81±9§,∥	37±9	17 (18)	42±7	16±8
4×250 kBq/kg ^227^Th-trastuzumab	4 weeks	8 (14)	93±18§,∥	48±18	12 (15)	29±4	4±4
4×5 µg trastuzumab	5 days/2 weeks	9 (12)	49±4	4±6	6 (8)	24±3	−1±4
Saline	–	18 (21)	45±4	0	8 (8)	25±2	0

* Number of tumors reaching 10-fold size (total number of tumors in therapy group).

† Days (mean ± standard error; estimation is limited to the largest time to reach endpoint within group if it is censored).

‡ Number of days delayed in reaching 10-fold increase in size compared to saline control group (mean ± standard error).

§ Significantly different from saline control group (p<0.001 for SKOV-3 and SKBR-3).

∥ Significantly different from trastuzumab control group (p<0.01 for SKOV-3 and p<0.001 for SKBR-3).

**Figure 4:**
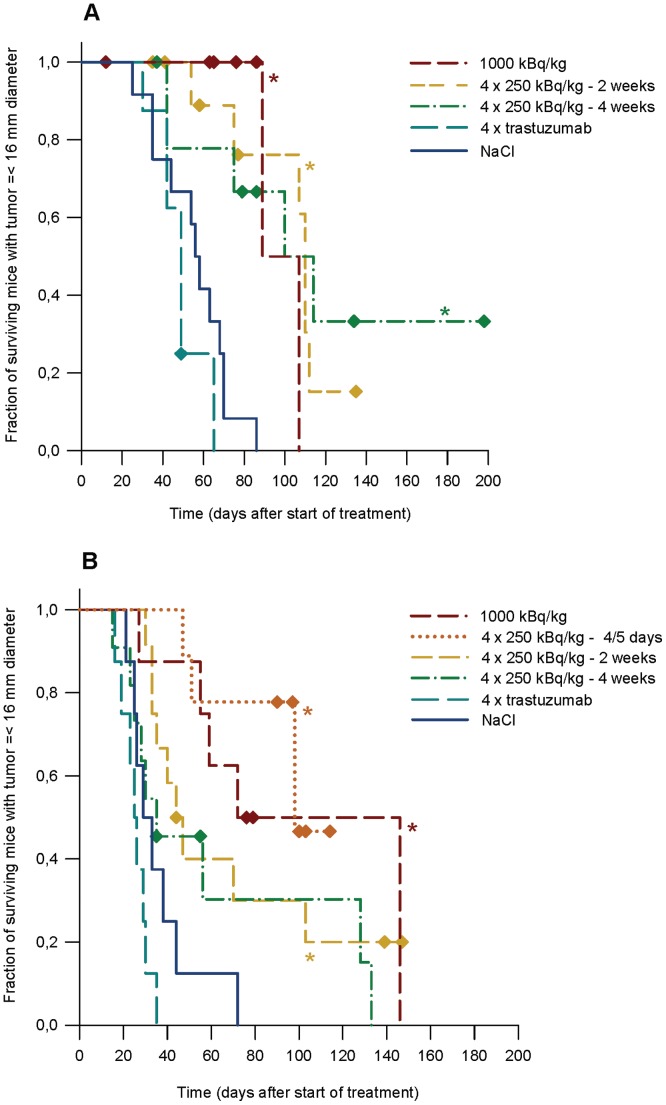
Survival with largest tumor diameter less than 16 mm. Survival of mice carrying SKOV-3 (A) and SKBR-3 (B) tumor xenografts as function of days after onset of therapy. The survival end point was tumor size of 16 mm diameter. Mice euthanized due to loss of weight were censored in each plot. Groups significantly different from saline (NaCl) control group is marked with an asterisk.

SKOV-3 tumor growth was initially more delayed in the group receiving a single dosage of 1000 kBq/kg body weight of ^227^Th-trastuzumab than in the groups given 250 kBq/kg body weight at intervals of 2 and 4 weeks, which seemed to follow the growth of the control groups (Figure 2B and 3A). However, growth of tumors given multiple TAT deviated from the control tumors after 2–3 injections. For SKOV-3 tumors treated with 1000 kBq/kg body weight, the initially decreased growth accelerated after about 40 days and the normalized average tumor size was about equal to the tumor size in the multiple injection groups after 65 days. All TAT groups showed delayed growth to 10-fold increase of SKOV-3 tumor size compared to saline and cold trastuzumab (p<0.001 and 0.01, respectively, Mantel-Cox Log Rank test, Table 1). No statistical differences were seen between the radioimmunotherapy groups. SKBR-3 tumor growth was delayed in the pilot study groups given 1000 kBq/kg body weight of ^227^Th-trastuzumab as single injection and 250 kBq/kg body weight at intervals of 4–5 days compared to the control groups (Figure 2A and 3B). The therapy groups given TAT at intervals of 2 and 4 weeks also showed a growth delay compared to control groups, although the difference was significant only for the 4 weeks interval group when compared to saline group (Figure 2C and 3B, p = 0.034 t-test, average tumor size 51 days post injection). Regrowth of individual tumors was seen also in the single injection and 4–5 days interval TAT groups after about 40 days. Table 1 shows that time to 10-doubling of tumor size after treatment with 1000 kBq/kg ^227^Th-trastuzumab as single injection and 4·250 kBq/kg ^227^Th-trastuzumab given at intervals of 4–5 days was significantly prolonged compared to control groups and therapy groups given 4·250 kBq/kg ^227^Th-trastuzumab at intervals of 2 and 4 weeks (p<0.001, Mantel-Cox Log Rank test). No statistically significant differences in growth to 10-fold increase were seen between groups given TAT at 2 and 4 week intervals and control groups for SKBR-3 tumors. However, data may indicate an effect in the 2 weeks interval group for some tumors, which was reflected in the slightly increased survival of mice in this group as compared with the control groups (Figure 4).

### Survival Analysis

Therapy response was also assessed by Kaplan Meyer survival analysis. Animals euthanized for other reasons than large tumor size were censored in the analysis. Survival data for SKOV-3 carrying mice showed that animals in all TAT therapy groups had improved survival compared to control groups (p<0.05, Mantel-Cox Log Rank test, Figure 4A), which was consistent with the above growth analysis. No significant differences between TAT groups were seen.

For the SKBR-3 animals, no significant differences were seen between multiple and single injection TAT groups (Figure 4B). However, therapy groups given 1000 kBq/kg ^227^Th-trastuzumab single injection and 4·250 kBq/kg ^227^Th-trastuzumab at intervals of 4–5 days and 2 weeks showed improved survival compared to control groups (p<0.01 for the first groups and p<0.05 for the latter, Mantel-Cox Log Rank test), while no significant improvement was seen for the 4 weeks interval group. The latter is consistent with the data in Table 1 and can be explained by the rapid growth of the SKBR-3 tumor xenografts compared to time between injections and the low dose rate of the ^227^Th. Most animals with SKBR-3 tumors in the groups with injections every 2^nd^ and 4^th^ week had to be sacrificed due to large tumor size before day 50. As a result, only 2 of 11 mice in the 4 weeks interval group received therapy injection number 3 at day 56 and most animals in the 2 weeks interval group had very large tumors when they received their 3^rd^ and 4^th^ therapy dose.

### Toxicity Assessment of ^227^Th-trastuzumab Multiple Injection Therapy by WBC Counts

**Figure 5:**
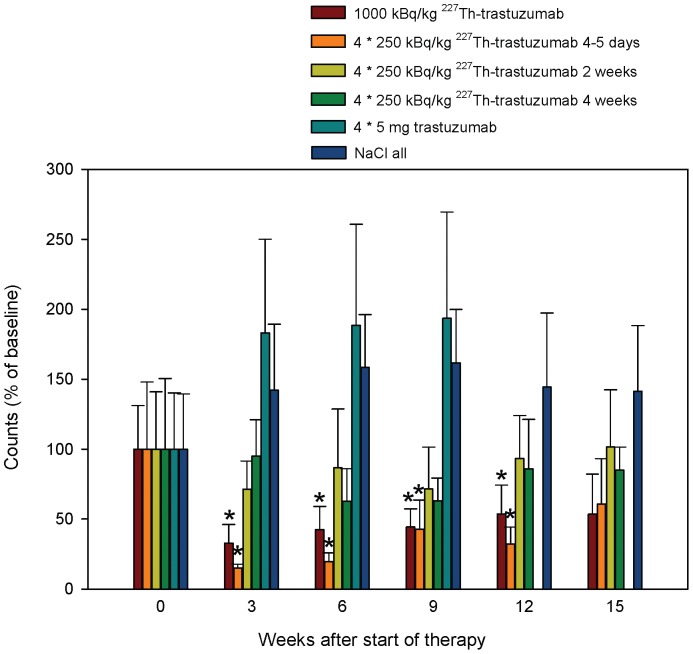
White blood cell counts as function of time. Normalized, pooled white blood cell counts for mice carrying SKOV-3 and SKBR-3 tumor xenografts as function of weeks after onset of therapy. In animals given saline (NaCl) data from animals without tumor are pooled with data from tumor-carrying animals. Whole blood from vena saphena lateralis was collected in EDTA-coated tubes and analyzed using an automated hematology analyzer (Scil Vet abc animal blood counter). Error bars are standard deviation. Results statistically significantly lower than baseline value are marked by an asterisk.

Data from mice bearing both tumor types were pooled in this analysis. For animals given saline, data for tumor-carrying animals were pooled with data from tumor free animals. A significant reduction in white blood cell counts from baseline level was seen 3–12 weeks after start of therapy in animals given 1000 kBq/kg ^227^Th-trastuzumab as single injection and 4·250 kBq/kg at intervals of 4–5 days (p≤0.002, Holm-Sidak Pairwise Multiple Comparison, Figure 5). No significant decrease was seen in the other TAT groups.

The number of red blood cells and platelets were counted, but no differences were seen after treatment for any of the tumor or therapy groups for these parameters.

### Toxicity Assessment by Clinical Chemistry Analysis

Clinical chemistry parameters were measured from full blood collected by heart puncture. Levels of urea, alanine aminotransferase (ALT), aspartate aminotransferase (AST) and alkaline phosphatase (ALP) were measured. For animals carrying SKOV-3 and SKBR-3 tumors no significant differences were seen between the different therapy groups (Table 2). For each clinical chemistry parameter, one or more samples had values well above the normal range; however, these seemed to be randomly distributed among the groups and were not sufficient to provide statistically significant differences (One Way ANOVA on Ranks and Dunn’s Pairwise Multiple Comparison).

**Table 2:** Overview of pooled clinical chemistry parameters in different therapy groups.

Parameters*		Urea (mmol/l)	ALT (U/l)	AST (U/l)	ALP (U/l)
Treatment group	Interval†	Mean (Range)	Mean (Range)	Mean (Range)	Mean (Range)
1000 kBq/kg ^227^Th-trastuzumab	–	12 (7–20)	67 (13–263)	447 (127–1148)	46 (20–261)
4×250 kBq/kg ^227^Th-trastuzumab	4–5 days	19 (7–89)	63 (23–169)	287 (123–1048)	34 (28–44)
4×250 kBq/kg ^227^Th-trastuzumab	2 weeks	12 (8–29)	45 (16–194)	197 (99–444)	35‡ (<20–64)
4×250 kBq/kg ^227^Th-trastuzumab	4 weeks	12 (7–37)	75 (13–332)	441 (40–3840)	47‡ (<20–252)
4×5 µg trastuzumab	5 days/2 weeks	12 (7–17)	30 (20–48)	229 (86–1176)	47 (30–63)
Saline	–	11 (4–15)	75 (16–415)	369 (83–1484)	53 (26–94)

* ALT: Alanine aminotransferase; AST: Aspartate aminotransferase; ALP: Alkaline phosphatase.

† Interval between therapy injections.

‡ ALP-values below detection limit included with the detection threshold value 20 U/l.

### Toxicity Assessment by Loss of Body Weight

**Figure 6:**
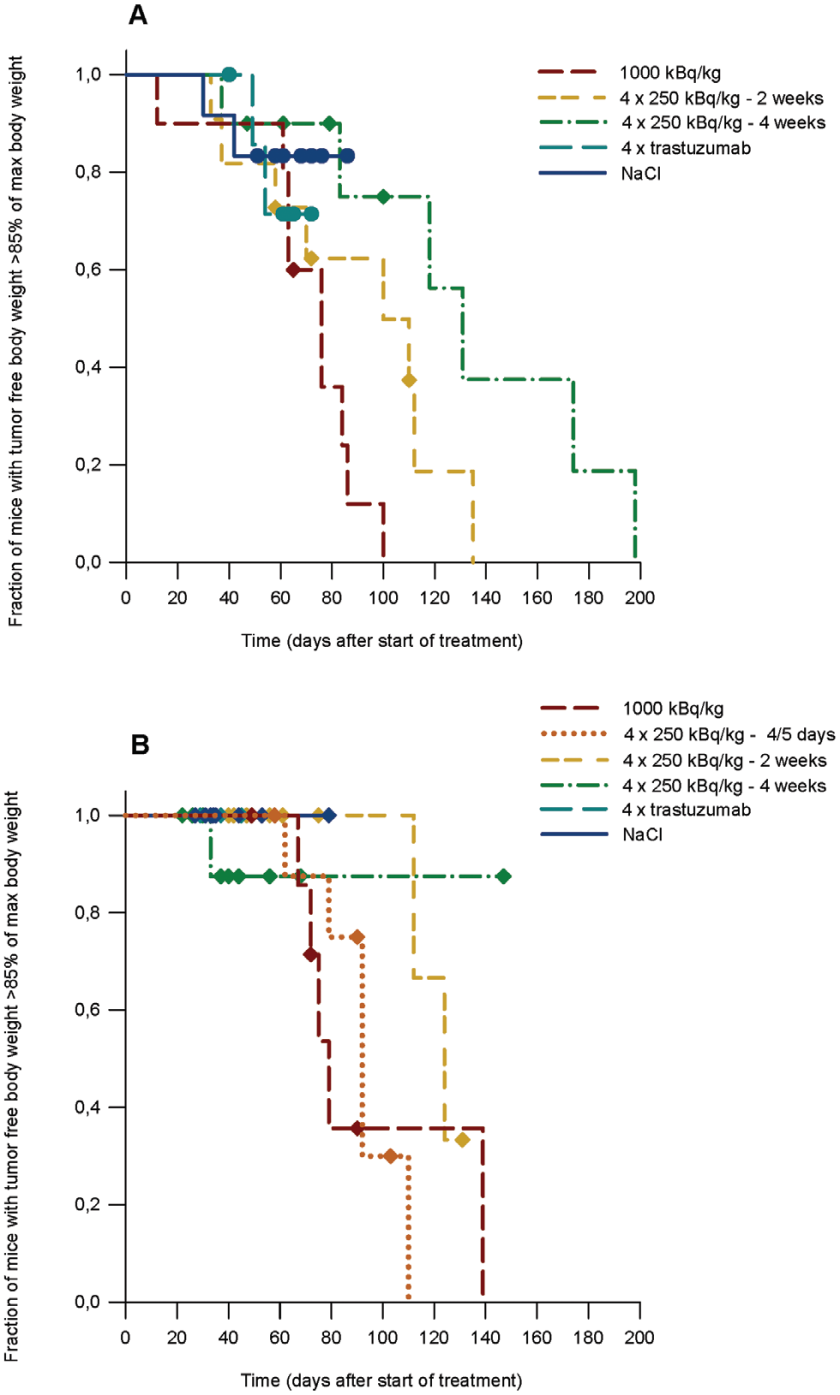
Survival without substantial loss of body weight. Survival of mice carrying SKOV-3 (A) and SKBR-3 (B) tumor xenografts as function of days after onset of therapy. The survival end point was loss of more than 15% of maximum body weight. Mice euthanized due to other reason than loss of weight were censored in each plot.

Survival without life threatening loss of body weight (defined as loss of body weight more than 15% of max tumor free body weight) was investigated as an important parameter of animal health (Figure 6). Tumor free body weight was calculated by subtracting calculated mass of tumors (volume multiplied by tumor density) from the measured body weight. Tumor density was measured in a selection of excised tumors and the mean value (0.87±0.09 kg/dm^3^) was used for calculations.

A high number of animals were euthanized due to loss of body weight in all SKOV-3 radioimmunotherapy groups, as seen in Figure 6A. The SKOV-3 group given 4·250 kBq/kg ^227^Th-trastuzumab at intervals of 4 weeks showed improved survival compared to animals given 1000 kBq/kg ^227^Th-trastuzumab as single injection (p<0.005, Mantel-Cox Log Rank test). Survival seemed improved also in the 2 week interval group, however this result was not statistically significant (p = 0.08). In the 4 week interval group 60% of the animals reached the endpoint of the survival analysis, compared to 73% and 90% for the 2 week interval and 1000 kBq/kg single injection groups, respectively. Events also occurred later in this group compared to the other therapy groups. Two animals in each of the SKOV-3 control groups reached the body weight endpoint (17% and 25% for saline and trastuzumab groups, respectively) indicating that the subcutaneous SKOV-3 tumor may be more harmful for the animals with regards to survival without life threatening loss of body weight than the SKBR-3 tumor model. The SKOV-3 tumors were in general more invasive than the SKBR-3 tumors in animals killed due to large tumor size.

For mice carrying SKBR-3 tumors, animals treated with 4·250 kBq/kg ^227^Th-trastuzumab given at 2 weeks interval showed improved survival to the group given therapy at intervals of 4–5 days (p<0.05, Mantel-Cox Log Rank test, Figure 6B). Only 2 animals (17%) in the 2 weeks interval group reached the body weight endpoint, however these animals lived longer than all the animals in the 4–5 day interval group and also longer than all animals but one in the single injection group. A few animals had a loss of body weight >15% of max tumor free body weight in all SKBR-3 groups except 1000 kBq/kg single injection and 4·250 kBq/kg ^227^Th-trastuzumab, 4–5 days interval (63% and 67% of the mice reached body weight endpoint, respectively). None of the mice in the SKBR-3 control groups had to be killed due to loss of body weight.

### Dosimetric Evaluations

**Figure 7:**
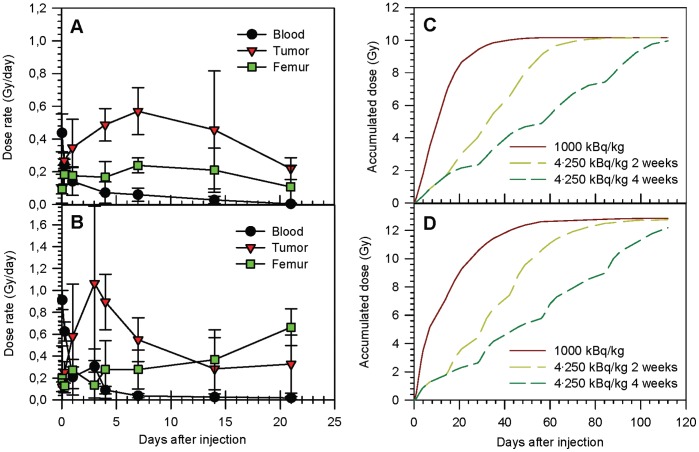
Dose rate and accumulated dose. Calculated dose rate based on measured radioactivity in selected organs in mice carrying SKOV-3 (A) and SKBR-3 (B) tumor xenografts. Dose rates are derived from biodistribution data presented previously [12], [13]. Cumulative dose calculations for SKOV-3 and SKBR-3 mice are shown for single injection with 1000 kBq/kg body weight and for multiple injections of 250 kBq/kg separated by 2 and 4 weeks in tumor (C, D). Calculations are based on the assumption that the shape of the dose rate curve is identical for every new injection.

An activity of 1000 kBq/kg of ^227^Th to the mouse corresponded to 0.5–1.0·10^9^ alpha-particle decays from ^227^Th per day and per gram of tumor at the maximum activity levels measured 3–7 days after injection of ^227^Th-trastuzumab (measurements were in the range 2.3–4.9 kBq/g after 3–7 days after injection of 400 kBq/kg, with large variations between individual tumors). When contribution from ^223^Ra and daughters were included in the absorbed radiation dose calculation, this corresponded to a maximum dose rate of 0.6 Gy/day in the SKOV-3 model and as high as 1.0 Gy/day in the SKBR-3 model. Dose rate curves for single injection SKOV-3 and SKBR-3 data were calculated based on biodistribution data previously published [12], [13]. Dose rate for tumor, femur and blood are shown in Figures 7A and 7B. The overall dose rate to tumor was kept on a relatively stable level by using 2 weeks interval between injections, while dose rate decreased considerably before next injection when allowing 4 weeks intervals, illustrated by the plateaus in accumulated tumor dose in Figure 7C, D.

## Discussion

Data presented in this report demonstrate that splitting the total activity of the radioimmunoconjugate ^227^Th-trastuzumab into several injections separated in time might reduce toxicity without reducing the therapeutic efficacy in HER2-expressing tumor xenograft models in athymic nude mice.

A dosage of 1000 kBq/kg ^227^Th-rituximab was previously shown to have good anti-tumor effect in CD20-overexpressing lymphoma xenografts with transient myelotoxicity, while the same dosage resulted in non-acceptable side effects in mice without tumor [14], [18]. However, since subcutaneous tumor xenograft will result in a lower % ID/g in the normal tissue, and since previous studies have shown no toxicity of 600 kBq/kg of ^227^Th-trastuzumab [12], 1000 kBq/kg of ^227^Th-trastuzumab was chosen as the single dosage in the present study. Furthermore, this dosage was split into four equal portions of 250 kBq/kg, which was expected to result in significantly reduced toxicity. Three separate intervals between dosages were investigated, as both the timeframes of damaging effect and recovery are difficult to predict. Whereas a single dosage of 250 kBq/kg ^227^Th-trastuzumab was considered too low to yield a therapeutic effect, repeated doses were predicted to yield an effect [12]. The toxicity and efficacy of the multiple dosing was compared to giving one dosage 1000 kBq/kg ^227^Th-trastuzumab.

Alpha particles will not hit all cells in the tumor due to a combination of the short range of alpha-particles, inhomogeneous HER2 distribution, limited penetration of the radioimmunoconjugate, the stochastic nature of alpha-particle radiation, and the long half-life of ^227^Th compared to the growth rate of tumor cells. Furthermore, previous studies suggest that the therapeutic effect of ^227^Th-TAT per dose unit is highest for the lowest doses, while for beta-RIT and external beam radiation the therapeutic effect per dose unit is constant [20]. The growth delay per dose unit was 5–15 days/Gy below 2 Gy for ^227^Th-TAT while at doses above 2 Gy it was around 3 days/Gy, which was comparable to beta-RIT and x-radiation [20]. This non-linearity may be due to the clustered DNA damage induced by high LET alpha particles as compared to predominantly single strand breaks induced by low LET beta particles or gamma radiation. Only a few clustered DNA lesions may be lethal to a cell and thus increasing the dose may lead to overkill and not necessarily a better therapeutic effect. Therefore, multiple dosing may be especially favorable for TAT using ^227^Th. Results on the SKOV-3 tumor model in the present study support that the therapeutic effect is at least as good with four dosages of 250 kBq as with one dosage of 1000 kBq/kg, as tumor growth delay for both multiple injection groups approached the single injection group after 65–70 days and as the time to 10-fold increase in tumor size was the longest for the multiple injection groups (Figure 2B, Table 1). Survival analysis also support this conclusion, as no statistically significant differences were seen between the multiple and single injection TAT groups, while all showed improved survival to the control groups (Figure 4A).

However, the therapeutic benefit of four dosages of 250 kBq/kg was not as obvious in the SKBR-3 xenografts. In the survival analysis, there were no significant differences in average survival between any of the multiple and single injection therapy groups, whereas a small delay was seen for the 2 and 4 weeks therapy groups in time to 10-doubling of tumor size. Already the pilot study indicated that the growth rate of the SKBR-3 tumors might be too rapid to be an optimal model due to the long half-life of ^227^Th. A few mice in the 4 weeks interval group lived long enough to have their 3^rd^ therapy injection, apparently mice with initially slowly growing tumors benefiting from the therapy. Interestingly, 2 mice in each of the 2 and 4 weeks interval groups had tumor diameters <16 mm close to or more than 130 days after start of therapy, which is considerably longer than any mouse in the control groups. The survival analysis takes account for events happening later than the tumor doubling analysis alone, and the statistical unit is the mouse, not the tumor, which explains the different results obtained with the two methods.

Several preclinical and clinical studies on fractionated or multiple RIT using β-emitting radionuclides combined with a wide range of antibodies indicate that fractionation can increase the therapeutic efficacy [17]. Clinical radioimmunotherapy using an ^90^Y-labeled anti-CD22 mAb or an ^131^I-labeled anti-CD20 mAb has been shown to yield high rates of overall responses and durable complete responses in relapsed/refractory Non-Hodgkin Lymphoma, when given as 2 injections separated by 2 and 8 weeks, respectively [21], [22]. No statistically difference in the efficacy between single dose and fractionated regime was seen in TAT with ^211^At-trastuzumab in intraperitoneally growing SKOV-3 and NIH:OVCAR-3 animal models [23], [24]. Together, the ^211^At results and our results suggest that there is no increased therapy effect of fractionation of TAT when the same total amount of activity is administered, although reduced adverse effects can be expected.

**Figure 8:**
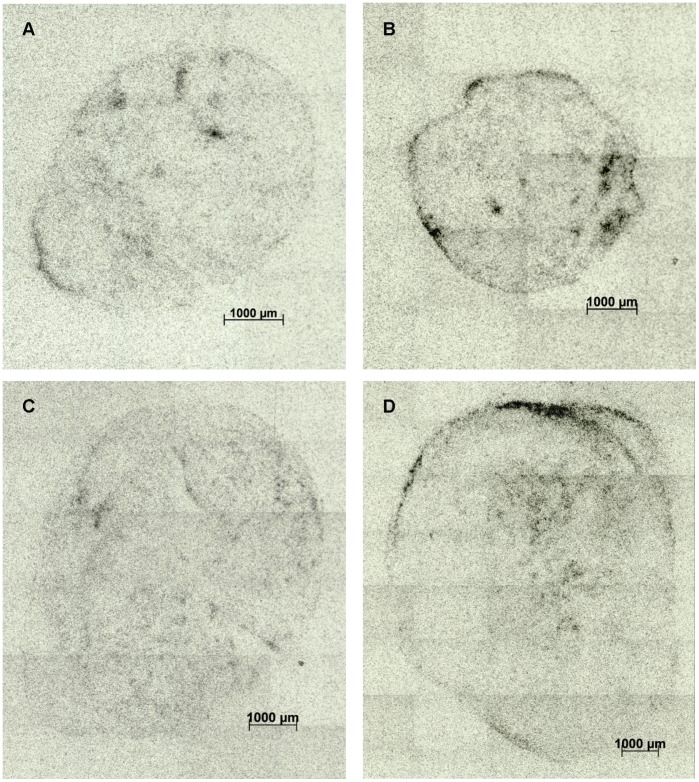
Autoradiography images of SKOV-3 and SKBR-3 tumors. Autoradiography images of the radioactivity distribution in 5 µm thick frozen tissue sections of SKOV-3 (A–B) and SKBR-3 (C–D) tumor xenografts from mice given 600 kBq/kg body weight ^227^Th-trastuzumab 8 days prior to tumor excition. Films were exposed for 7 days in −80°C prior to development.

The dose contribution to the tumor in TAT is always limited by the antibody distribution in the tumor. Firstly, mAbs are large molecules showing slow diffusion and poor penetration. Secondly, high binding affinity to target may contribute to reduce distribution of the mAb according to the “binding-site barrier” hypothesis [25]. Therefore saturation of the antigen sites in the perivascular regions may be required in order for the mAb to penetrate deeper into the tumor. The average penetration depth of trastuzumab into SKOV-3 tumors was measured by Rudnick et al. to be 37 µm from the blood vessels 5 days after i.p. injection of 500 µg [26]. Autoradiography images of typical SKOV-3 and SKBR-3 tumors given ^227^Th-trastuzumab are shown in Figure 8. Inhomogeneous distribution of the radioimmunoconjugate across tumors can be seen. The limited penetration of the radioimmunoconjugate together with the low range of the alpha-particles might explain why TAT fractionated into small dosages does not seem to give as much benefit as fractionated therapy with longer range β-particles in solid tumors. Fractionated TAT was however reported to be successful in intraperitoneally growing disseminated NIH:OVCAR-3 ovarian cancer xenografts when using a higher activity closer to the maximum tolerated dose per fraction [27].

Dose rate calculations based on earlier studies of SKOV-3 and SKBR-3 xenografts in mice indicated that the dynamic tumor uptake might be different in the two tumor models, resulting in different total accumulated dose. For tumor tissue this approach is an oversimplification of the real situation as we know that activity is not evenly distributed throughout the tumor. Further, antigen expression might be different in the two tumor models. Previous *in vitro* studies showed that SKBR-3 cells had more antigen sites than SKOV-3 cells; however the antigen expression showed a broader distribution in the SKOV-3 population [11]. Regarding contribution from free ^223^Ra, it is assumed that the alpha particles emitted from tumor-localized ^223^Ra are mainly absorbed by the tumor cells due to the high cell density. Biodistribution data from previous studies [12], [13] may indicate that ^223^Ra is less likely to escape from SKBR-3 tumors compared to SKOV-3 tumors, contributing to a higher total dose in the SKBR-3 tumors as seen in Figure 8. However, large variations in dose to individual tumors were seen, making conclusions difficult. In the bone marrow, however, the situation is different as free ^223^Ra is not homogeneously distributed but located mainly on bone surface. Due to the short alpha-particle range, the ^223^Ra is less likely to harm the cells of the bone marrow [14]. When comparing the results to *in vitro* studies in the same cell lines, the mean total dose per cell from cell-bound ^227^Th-trastuzumab needed to kill 10-fold of tumor cells was about 2 Gy both for SKOV-3 and SKBR-3 cell lines, however the SKOV-3 cells were found to be a bit more radiosensitive than the SKBR-3 cells [11]. This fact may partly explain the better response in the SKOV-3 tumor xenografts.

The data suggest that splitting the dose can alleviate red marrow toxicity, which might be the dose limiting factor in ^227^Th-TAT, due to relocation of the long lived daughter nuclide ^223^Ra to bone [12], [14]. This result corresponds with the reported results from a study by Elgqvist et al, where myelotoxicity was reduced in fractionated TAT with ^211^At [24]. No statistically significant differences were seen in the clinical chemistry analysis, a few samples with values above the normal range seemed to be randomly distributed among the therapy groups.

Loss of body weight was used as another indicator of toxicity. In the groups given fractionated therapy, fewer animals were killed due to loss of body weight, or later than in the group given 1000 kBq/kg ^227^Th-trastuzumab. An exception is the group given fractions at intervals 4–5 days. The toxicity in this group seemed to be at least as high as for 1000 kBq/kg single injection, possibly indicating that the interval is too short to allow for washout of radioactivity in blood between the injections, thus keeping the radioactivity level in blood and bone marrow high over a prolonged period. The serum half-life of ^227^Th-trastuzumab could be estimated from previously published biodistribution data to be 7–10 hours in the initial phase right after injection, and 2–5 days later in the study, when 0.4–0.6 µg trastuzumab/kg body weight was injected [12], [13].

There is an inherent problem in studying fractionated radioimmunetherapy in mice with rapidly growing tumors, especially when using long-lived, low-dose-rate emitting radionuclides such as ^227^Th. A compromise with regards to dosage and intervals will need to be made taking the half-life of the radionuclide, tumor growth and time to white blood cell recovery into account. In this case the myeloid toxicity of the bone-seeking daughter radionuclide ^223^Ra needs to be considered, as well as the half-life of the ^227^Th-mAb conjugate in circulation, as it is likely that the major proportion of ^223^Ra in bone derives from the decay of circulating mAb-bound ^227^Th [12], [14]. Due to the long half-life of ^223^Ra (11.4 days), several weeks between fractions might be optimal. This is however suboptimal for fast growing tumors as proliferation will occur in cells not hit by alpha-particles. Sub-toxic dosage levels should however be possible to administer with shorter intervals as discussed by DeNardo [17]. We suggest that fractionated therapy in humans with longer intervals is even more beneficial with regards to side effects. For example, an interval of 4 weeks has been used in clinical trials of intravenous administrations of 50 kBq/kg body weight of ^223^ Ra with minimum myelotoxicity, after up to six consecutive injections in prostate cancer patients suffering from bone metastases [28], [29].

### Conclusions/Significance

The study indicates that the same concentration of radioactivity split into several fractions may reduce toxicity of ^227^Th-radioimmunotherapy while the therapeutic effect is maintained. Thus, it might be possible to increase the cumulative absorbed radiation dose to tumor with acceptable toxicity by fractionation of the dosage.

Thorium-227 TAT may, however, not be convenient for fractionated use in very rapidly growing tumors due to the low dose rate by which the dose is delivered to the tumor tissue.

## References

[1] Ferlay J, Shin HR, Bray F, Forman D, Mathers C, et al (2010) Estimates of worldwide burden of cancer in 2008: GLOBOCAN 2008. Int J Cancer 127: 2893–2917. 21351269 10.1002/ijc.25516

[2] Siegel R, Ward E, Brawley O, Jemal A (2011) Cancer statistics, 2011: the impact of eliminating socioeconomic and racial disparities on premature cancer deaths. CA Cancer J Clin 61: 212–236. 21685461 10.3322/caac.20121

[3] Steger GG, Abrahamova J, Bacanu F, Brincat S, Brize A, et al (2010) Current standards in the treatment of metastatic breast cancer with focus on Lapatinib: a review by a Central European Consensus Panel. Wien Klin Wochenschr 122: 368–379. 20549373 10.1007/s00508-010-1373-6

[4] Wolff AC, Hammond ME, Schwartz JN, Hagerty KL, Allred DC, et al (2007) American Society of Clinical Oncology/College of American Pathologists guideline recommendations for human epidermal growth factor receptor 2 testing in breast cancer. Arch Pathol Lab Med 131: 18–43. 19548375 10.5858/2007-131-18-ASOCCO

[5] Verri E, Guglielmini P, Puntoni M, Perdelli L, Papadia A, et al (2005) HER2/neu oncoprotein overexpression in epithelial ovarian cancer: evaluation of its prevalence and prognostic significance. Clinical study. Oncology 68: 154–161. 16020953 10.1159/000086958

[6] Nahta R, Esteva FJ (2006) Herceptin: mechanisms of action and resistance. Cancer Lett 232: 123–138. 16458110 10.1016/j.canlet.2005.01.041

[7] Nahta R, Esteva FJ (2007) Trastuzumab: triumphs and tribulations. Oncogene 26: 3637–3643. 17530017 10.1038/sj.onc.1210379

[8] Milenic DE, Wong KJ, Baidoo KE, Nayak TK, Regino CA, et al (2010) Targeting HER2: a report on the in vitro and in vivo pre-clinical data supporting trastuzumab as a radioimmunoconjugate for clinical trials. MAbs 2: 550–564. 20716957 10.4161/mabs.2.5.13054 PMC2958576

[9] Sgouros G, Ballangrud AM, Jurcic JG, McDevitt MR, Humm JL, et al (1999) Pharmacokinetics and dosimetry of an alpha-particle emitter labeled antibody: 213Bi-HuM195 (anti-CD33) in patients with leukemia. J Nucl Med 40: 1935–1946. 10565792

[10] Larsen RH, Borrebaek J, Dahle J, Melhus KB, Krogh C, et al (2007) Preparation of TH227-labeled radioimmunoconjugates, assessment of serum stability and antigen binding ability. Cancer Biother Radiopharm 22: 431–437. 17651051 10.1089/cbr.2006.321

[11] Heyerdahl H, Krogh C, Borrebaek J, Larsen A, Dahle J (2011) Treatment of HER2-expressing breast cancer and ovarian cancer cells with alpha particle-emitting 227Th-trastuzumab. Int J Radiat Oncol Biol Phys 79: 563–570. 21195878 10.1016/j.ijrobp.2010.08.038

[12] Abbas N, Heyerdahl H, Bruland O, Borrebaek J, Nesland J, et al (2011) Experimental alpha-particle radioimmunotherapy of breast cancer using 227Th-labeled p-benzyl-DOTA-trastuzumab. EJNMMI Research 1: 18. 22214432 10.1186/2191-219X-1-18 PMC3250964

[13] Abbas N, Bruland O, Brevik EM, Dahle J (2012) Preclinical evaluation of ^227^Th- and ^177^Lu-labeled-trastuzumab in mice with HER-2 positive ovarian cancer xenografts. Nucl Med Commun In Press. 10.1097/MNM.0b013e328354df7c 22643311

[14] Dahle J, Jonasdottir TJ, Heyerdahl H, Nesland JM, Borrebaek J, et al (2010) Assessment of long-term radiotoxicity after treatment with the low-dose-rate alpha-particle-emitting radioimmunoconjugate (227)Th-rituximab. Eur J Nucl Med Mol Imaging 37: 93–102. 19593562 10.1007/s00259-009-1197-7

[15] Kennel SJ (1992) Effects of target antigen competition on distribution of monoclonal antibody to solid tumors. Cancer Res 52: 1284–1290. 1737391

[16] Schlom J, Molinolo A, Simpson JF, Siler K, Roselli M, et al (1990) Advantage of dose fractionation in monoclonal antibody-targeted radioimmunotherapy. J Natl Cancer Inst 82: 763–771. 2182892 10.1093/jnci/82.9.763

[17] DeNardo GL, Schlom J, Buchsbaum DJ, Meredith RF, O’Donoghue JA, et al (2002) Rationales, evidence, and design considerations for fractionated radioimmunotherapy. Cancer 94: 1332–1348. 11877764 10.1002/cncr.10304

[18] Dahle J, Borrebaek J, Jonasdottir TJ, Hjelmerud AK, Melhus KB, et al (2007) Targeted cancer therapy with a novel low-dose rate alpha-emitting radioimmunoconjugate. Blood 110: 2049–2056. 17536011 10.1182/blood-2007-01-066803

[19] Lindmo T, Boven E, Cuttitta F, Fedorko J, Bunn PA Jr (1984) Determination of the immunoreactive fraction of radiolabeled monoclonal antibodies by linear extrapolation to binding at infinite antigen excess. J Immunol Methods 72: 77–89. 6086763 10.1016/0022-1759(84)90435-6

[20] Dahle J, Bruland OS, Larsen RH (2008) Relative biologic effects of low-dose-rate alpha-emitting 227Th-rituximab and beta-emitting 90Y-tiuexetan-ibritumomab versus external beam X-radiation. Int J Radiat Oncol Biol Phys 72: 186–192. 18722269 10.1016/j.ijrobp.2008.05.029

[21] Morschhauser F, Kraeber-Bodere F, Wegener WA, Harousseau JL, Petillon MO, et al (2010) High rates of durable responses with anti-CD22 fractionated radioimmunotherapy: results of a multicenter, phase I/II study in non-Hodgkin’s lymphoma. J Clin Oncol 28: 3709–3716. 20625137 10.1200/JCO.2009.27.7863

[22] Illidge TM, Bayne M, Brown NS, Chilton S, Cragg MS, et al (2009) Phase 1/2 study of fractionated (131)I-rituximab in low-grade B-cell lymphoma: the effect of prior rituximab dosing and tumor burden on subsequent radioimmunotherapy. Blood 113: 1412–1421. 19074729 10.1182/blood-2008-08-175653

[23] Palm S, Back T, Claesson I, Danielsson A, Elgqvist J, et al (2007) Therapeutic efficacy of astatine-211-labeled trastuzumab on radioresistant SKOV-3 tumors in nude mice. Int J Radiat Oncol Biol Phys 69: 572–579. 17869670 10.1016/j.ijrobp.2007.06.023

[24] Elgqvist J, Andersson H, Back T, Claesson I, Hultborn R, et al (2006) Fractionated radioimmunotherapy of intraperitoneally growing ovarian cancer in nude mice with 211At-MX35 F(ab’)2: therapeutic efficacy and myelotoxicity. Nucl Med Biol 33: 1065–1072. 17127181 10.1016/j.nucmedbio.2006.07.009

[25] Fujimori K, Covell DG, Fletcher JE, Weinstein JN (1990) A modeling analysis of monoclonal antibody percolation through tumors: a binding-site barrier. J Nucl Med 31: 1191–1198. 2362198

[26] Rudnick SI, Lou J, Shaller CC, Tang Y, Klein-Szanto AJ, et al (2011) Influence of affinity and antigen internalization on the uptake and penetration of Anti-HER2 antibodies in solid tumors. Cancer Res 71: 2250–2259. 21406401 10.1158/0008-5472.CAN-10-2277 PMC3077882

[27] Elgqvist J, Andersson H, Jensen H, Kahu H, Lindegren S, et al (2010) Repeated Intraperitoneal alpha-Radioimmunotherapy of Ovarian Cancer in Mice. J Oncol 2010: 394913. 19859581 10.1155/2010/394913 PMC2766502

[28] Nilsson S, Franzen L, Parker C, Tyrrell C, Blom R, et al (2007) Bone-targeted radium-223 in symptomatic, hormone-refractory prostate cancer: a randomised, multicentre, placebo-controlled phase II study. Lancet Oncol 8: 587–594. 17544845 10.1016/S1470-2045(07)70147-X

[29] Parker C, Heinrich D, O’Sullivan JM, Fosså S, Chodacki A, et al (2011) Overall Survival Benefit of Radium-223 Chloride (Alpharadin) in the Treatment of Patients with Symptomatic Bone Metastases in Castration-resistant Prostate Cancer (CRPC): a Phase III Randomized Trial (ALSYMPCA). Eur J Cancer 47(suppl2): 3.

